# Efficacy and safety of chimeric antigen receptor T cells targeting BCMA and GPRC5D in relapsed or refractory multiple myeloma

**DOI:** 10.3389/fimmu.2024.1466443

**Published:** 2024-12-23

**Authors:** Xu Yang, Feiqing Wang, Xiaoshuang Yuan, Bo Yang, Juan Chen, Jinyang Cheng, Guangyang Liu, Dongxin Tang, Xiao Xu, Sanbin Wang, Zhixu He, Yang Liu, Yanju Li

**Affiliations:** ^1^ Clinical Medical Research Center, The First Affiliated Hospital of Guizhou University of Traditional Chinese Medicine, Guiyang, Guizhou, China; ^2^ Academy of Medical Engineering and Translational Medicine, Tianjin University, Tianjin, China; ^3^ Department of Hematology, Affiliated Hospital of Guizhou Medical University, Guiyang, Guizhou, China; ^4^ Fourth Medical Center, People’s Liberation Army General Hospital, Beijing, China; ^5^ Department of Hematology, The 920th Hospital of Joint Logistics Support Force, Kunming, Yunnan, China; ^6^ Key Laboratory of Adult Stem Cell Translational Research, Chinese Academy of Medical Sciences, Guizhou Medical University, Guiyang, Guizhou, China

**Keywords:** B-cell maturation antigen, G protein-coupled receptor, class C group 5 member D, car-T, relapsed or refractory multiple myeloma

## Abstract

**Background:**

Clinical studies have demonstrated the high efficacy of using chimeric antigen receptor (CAR)-T cells targeting B-cell maturation antigen (BCMA) and orphan G protein-coupled receptor, class C group 5 member D (GPRC5D) to treat relapsed or refractory multiple myeloma (RRMM). In this study, we compared the efficacy and safety of BCMA CAR-T-cell therapy (BCMA CAR-T) and GPRC5D CAR T-cell therapy (GPRC5D CAR-T) in patients with RRMM.

**Methods:**

We retrieved and included eligible clinical trials of BCMA or GPRC5D CAR-T for RRMM patients. The primary outcomes for efficacy were overall response rate (ORR), complete response rate (CRR), minimal residual disease (MRD) negativity, and relapse rate. The primary outcomes for safety were cytokine release syndrome (CRS) and immune effector cell-associated neurotoxicity syndrome (ICANS).

**Results:**

We incorporated 18 early-phase, single-arm clinical trials, which included 503 and 133 patients receiving BCMA CAR-T and GPRC5D CAR-T, respectively. For the GPRC5D CAR-T cohort, the estimated ORR, CRR, MRD negativity rate, and relapse rate were found to be 89.8% [95% confidence interval (CI), 82.8%–96.9%], 50.5% (95% CI, 38.0%–62.9%), 78.8% (95% CI, 53.0%–100%), and 26.0% (95% CI, 7.4%–44.6%), respectively. In the BCMA CAR-T group, the ORR was 76.3% (95% CI, 67.9%–84.7%), the CRR was 34.3% (95% CI, 25.9%–42.7%), the MRD negativity rate was 76.5% (95% CI, 63.1%–90.0%), and the recurrence rate was 57.3% (95% CI, 47.7%–66.9%). These values were significantly lower than those observed in the GPRC5D CAR-T cohort. Both BCMA and GPRC5D CAR-T demonstrated acceptable safety. The estimated incidence of BCMA CAR-T resulting in grade 3–5 CRS and ICANS was only 5.4% (95% CI, 2.0%–10.4%) and 3.3% (95% CI, 0.6%–8.0%), respectively. The estimated incidence of GPRC5D CAR-T resulting in grade 3–5 CRS and ICANS was only 1.6% (95% CI, 0.0%–6.5%) and 2.7% (95% CI, 0.7%–6.2%), respectively.

**Conclusion:**

GPRC5D CAR-T potentially demonstrates enhanced effectiveness relative to BCMA CAR-T in treating patients with RRMM. Therefore, GPRC5D CAR-T can be regarded as the preferred therapeutic option for RRMM, particularly among patients who have undergone relapse subsequent to BCMA CAR-T treatment.

## Introduction

Multiple myeloma (MM) is a malignant plasma cell neoplasm, constituting approximately 10% of all hematological malignancies (1, 2). Despite several therapeutic advances, MM still remains, for most patients, incurable (3). Nevertheless, with the advent of novel therapeutic agents, such as proteasome inhibitors (PIs), immunomodulatory drugs (IMiDs), anti-CD38 monoclonal antibodies, selective nuclear export protein inhibitors (SINEs), and T-cell–redirected bispecific antibodies, over the past decade, the survival outcomes of patients with MM have improved considerably (4–7). However, nearly all patients eventually experience relapse due to drug resistance (8). Particularly concerning are those with relapsed or refractory multiple myeloma (RRMM) and individuals presenting with high-risk cytogenetic features or extramedullary disease (EMD), who exhibit a dismal prognosis (9, 10). Consequently, there is an urgent necessity for innovative therapeutic approaches that target RRMM.

In preclinical evaluations, therapies based on chimeric antigen receptor (CAR)-T cells have demonstrated high efficacy against MM, particularly RRMM (11, 12). B-cell maturation antigen (BCMA) is consistently expressed on MM cells but is absent from normal tissues or plasma cells (13). Brudno et al. conducted the first trial on BCMA-targeted CAR-T therapy (hereinafter referred to as BCMA CAR-T) and reported a high response rate in patients with RRMM (14). Currently, idecabtagene vicleucel (ide-cel, bb2121) and ciltacabtagene autoleucel (cilta-cel) are two BCMA CAR-T modalities approved for adult RRMM patients with at least two prior lines of therapy for ide-cel and one prior line of therapy for cilta-cel, including a PI, an IMiD, and an anti-CD38 monoclonal antibody. Nevertheless, neither modality has demonstrated sustained survival benefits in this patient population, with most patients eventually experiencing relapse (15–18).

In the realm of MM treatment, orphan G protein-coupled receptor class C group 5 member D (GPRC5D) has emerged as a promising alternative target for CAR-T cell therapy (19). This receptor is not only present in the bone marrow plasma cells of MM patients but is also expressed in MM cell lines (19, 20). We recently performed an early dose escalation trial, MCARH109, which presents the first formally published results regarding the activity of GPRC5D-targeted CAR-T cells (hereinafter: GPRC5D CAR-T) in patients with RRMM (including those previously treated with BCMA CAR-T) (21). The results confirmed that GPRC5D is an effective immunotherapeutic target for CAR-T therapy in RRMM.

Although both BCMA CAR-T and GPRC5D CAR-T may effectively resolve RRMM, no study has compared their efficacies in these patients. Furthermore, most studies thus far have included a small sample size and lacked sufficient validation. Therefore, in this systematic review and meta-analysis, we compared the efficacies and safety of BCMA and GPRC5D CAR-T therapies in RRMM to provide a theoretical basis for the clinical treatment of the malignancy.

## Methods

### Data sources and search strategy

We searched several publication databases, including PubMed, ScienceDirect, Embase, and Medline, for eligible studies published until December 2023. Only clinical trials published in English and registered at Clinicaltrials.gov (NCT number) or in the Chinese Clinical Trial Registry (ChiCTR number) were included. The following English search terms were used for this search: “B-cell maturation antigen” or “BCMA”; “chimeric antigen receptor” or “CAR”; “G protein-coupled receptor, family C, group 5, member D” or “GPRCD”; “Relapse or Refractory Multiple Myeloma”; and “clinical trials.” We also included eligible full articles or abstracts presented at the annual scientific meetings of the American Society of Clinical Oncology (ASCO), American Society of Hematology (ASH), and European Association of Hematology (EHA). Patient data were extracted only from the obtained articles; no additional requests for original patient data were made by any of the authors.

### Inclusion and exclusion criteria

We only included studies that (1) were published in English, (2) were clinical trials on BCMA or GPRC5D CAR-T, and (3) included patients with RRMM regardless of their age or sex. In contrast, we excluded studies that did not (1) assess the effects of BCMA or GPRC5D CAR-T in patients with RRMM, (2) provide data required for meta-analysis (e.g., total patient number, CAR-T efficacy, and adverse reactions), or (3) use a clinical trial design (e.g., review, case report, or animal study).

### Data extraction

The literature search, abstract and full-text review, and data collection were independently performed by two authors, followed by a cross-review for data collection accuracy. The primary outcome measures were overall response rate (ORR), complete response rate (CRR), minimal residual disease (MRD) negativity, relapse rate, and CAR-T-related toxicity [i.e., cytokine release syndrome (CRS) and immune effector cell-associated neurotoxicity syndrome (ICANS)]. We defined ORR according to the International Myeloma Working Group criteria as the total of (strict) complete responses and (very good) partial responses (22). For each study, we collected the following information: authors, year of publication, median patient age, patient number, line of prior treatment, median follow-up duration, treatment targets, efficacy outcome measures (ORR, CRR, MRD negativity, and relapse rate), and safety outcome measures (CRS and ICANS incidence).

### Risk of bias and quality evaluation

We used the Methodological Index for Non-Randomized Studies (MINORS) scale to evaluate the methodological quality of each study (23). Since none of the studies included a control group, we only used eight MINORS items, with the maximum score for each study set at 16. We assessed the certainty of the body of evidence in the domains of risk of bias, originality, imprecision, inconsistency, and publication bias using hierarchical methods for all included studies. According to the quality of evidence recorded in the GRADE system, we used the following evidence levels: high, medium, low, and very low (24).

### Statistical analysis

Because of the diversity among the included studies, we used random-effects models to obtain outcome rates along with their 95% confidence intervals (CIs). Subgroup analyses were performed to assess differences between study groups, with proportions pooled using the random-effect models (DerSimonian–Laird). Freeman–Tukey double inverse sine transformation was used when the data did not follow a normal distribution. Interassay heterogeneity was measured using the *I^2^* statistic (i.e., the percentage of studied variation due to heterogeneity rather than chance). All analyses were performed using R (version 4.3.2), and *p* < 0.05 indicated statistical significance.

## Results

### Literature search results

**Figure 1:**
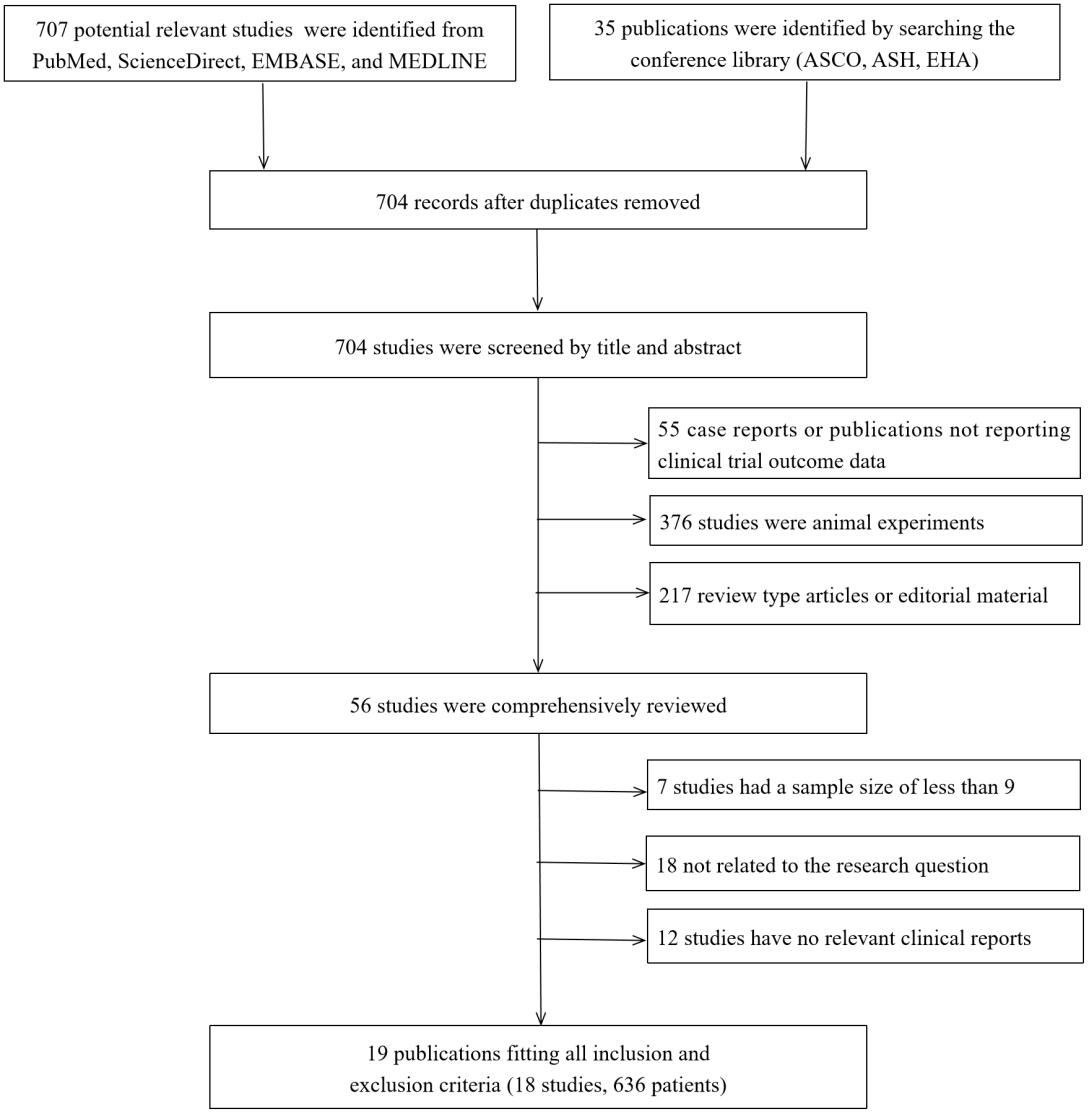
Search strategy and study selection. ASCO, American Society of Clinical Oncology; ASH, American Society of Hematology; EHA, European Hematology Association.

Our initial search yielded 707 abstracts, clinical studies, case studies, and other publications. Of these, only 19 studies were eligible (14, 21, 25–41). However, because two of these studies (40, 41) had an identical clinical trial number (NCT04674813), 18 studies were finally included. All of these studies were early-stage single-arm clinical trials. In these trials, 503 patients were administered BCMA CAR-T, while 133 received GPRC5D CAR-T. The flow of the study selection process is presented in **Figure 1**.

### Characteristics and MINORS grades of the included studies

**Table 1** presents the clinical data from the included studies. All trials were published between 2018 and 2023, and their sample sizes ranged from 9 to 128. Of all 18 included studies, 14 and 4 focused on BCMA and GPRC5D CAR-T, respectively. In the intervention protocols, the dosage ranged from 7.5 × 10^5^ to 8.0 × 10^8^ CAR-T cells/kg.

**Table 1:** Characteristics of the selected studies.

Study ID	No. Studies	Age	Line of previous treatment regimen	CAR T target	Median follow-up time	ORR(%)	CRR(%)	MRD negative(%)	Relapse(%)	Serious CRS(%)	Serious ICANS(%)	MINORS grade
Minakata2023 (25)	9	54	4	BCMA	12.9 Months	89%	55.6%	66.7%	25.0%	0	0	13
Asherie 2023 (26)	20	62	6	BCMA	4.5 Months	75.0%	50.0%	40.0%	53.3%	0	0	14
Mailankody 2023 (27)	43	66.3	5	BCMA	10.2 Months	55.8%	35.3%	92.9%	54.2%	2.3%	0	13
Qu 2022 (28)	31	65	5	BCMA	9.4 Months	87.1%	45.2%	93.8%	NA	9.7%	3.2%	13
Du 2022 (29)	49	66	4	BCMA	≥1 Months	77.6%	46.9%	42.9%	68.4%	6.1%	NA	12
Munshi 2021 (30)	128	67	5	BCMA	13.3 Months	73.4%	32.8%	78.6%	NA	5.5%	3.1%	13
Raje 2021 (31)	33	66	7	BCMA	11.3 Months	84.8%	45.5%	83.3%	53.6%	6.1%	3.0%	13
Alsina 2020 (32)	46	62	6	BCMA	8.5 Months	54.3%	17.4%	NA	NA	4.3%	6.5%	13
An 2020 (33)	21	60	4	BCMA	182 Days	95.2%	28.6%	NA	NA	5%	NA	12
Kumar 2020 (34)	14	59	6	BCMA	4.5 months	100%	40%	91.7%	NA	0	0	12
Fu 2019 (35)	44	NA	NA	BCMA	≥1 Months	79.65	40.9%	36.4%	NA	6.8%	NA	11
Cohen 2019 (36)	25	58	5	BCMA	NA	48.0%	8%	NA	75.0%	32%	8.0%	12
Brudno 2018 (14)	26	56.5	9.5	BCMA	NA	57.7%	11.5%	NA	53.8%	37.5%	37.5%	12
Liu 2018 (37)	14	NA	NA	BCMA	≥1 Months	78.6%	50.0%	64.3%	NA	0	7.1%	11
Xia 2023 (38)	44	63	5	GPRC5D	10 Months	90.9%	63.6%	78.8%	16.7%	0	2.3%	13
Zhang 2023 (39)	10	64	5.5	GPRC5D	9 Months	100%	60.0%	100%	20.0%	0	0	13
Mailankody 2023 (27)	17	58.5	4	GPRC5D	10.1 Months	70.6%	35.3%	52.9%	50.0%	5.9%	5.9%	12
Bal 2023 (40, 41)	64	NA	NA	GPRC5D	5.9 Months	87.7%	45.2%	NA	NA	4.3%	2.9%	11

BCMA, B cell membrane antigen; GPRC5D, G protein-coupled receptor, class C group 5 member D; ORR, overall response rate; CRR, complete response rate; MRD, minimal residual lesion negative; CRS, Cytokine release syndrome; ICANS, Immune effector cell-associated neurotoxicity syndrome.

NA, not available.

### Response rates in RRMM patients treated with BCMA and GPRC5D CAR-T

**Figure 2:**
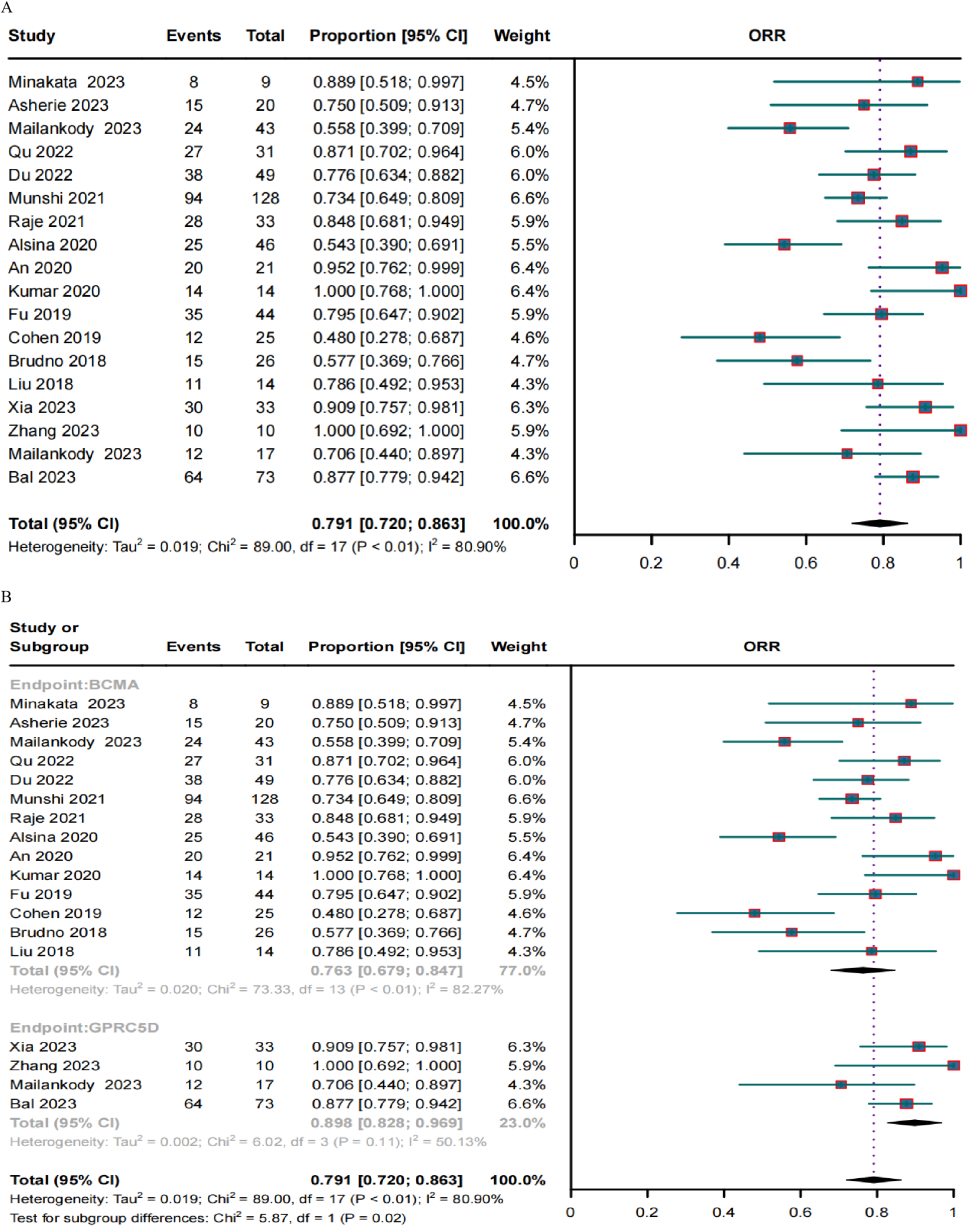
Forest plots of **(A)** pooled and **(B)** subgroup ORR data for BCMA and GPRC5D CAR-T.

**Figure 3:**
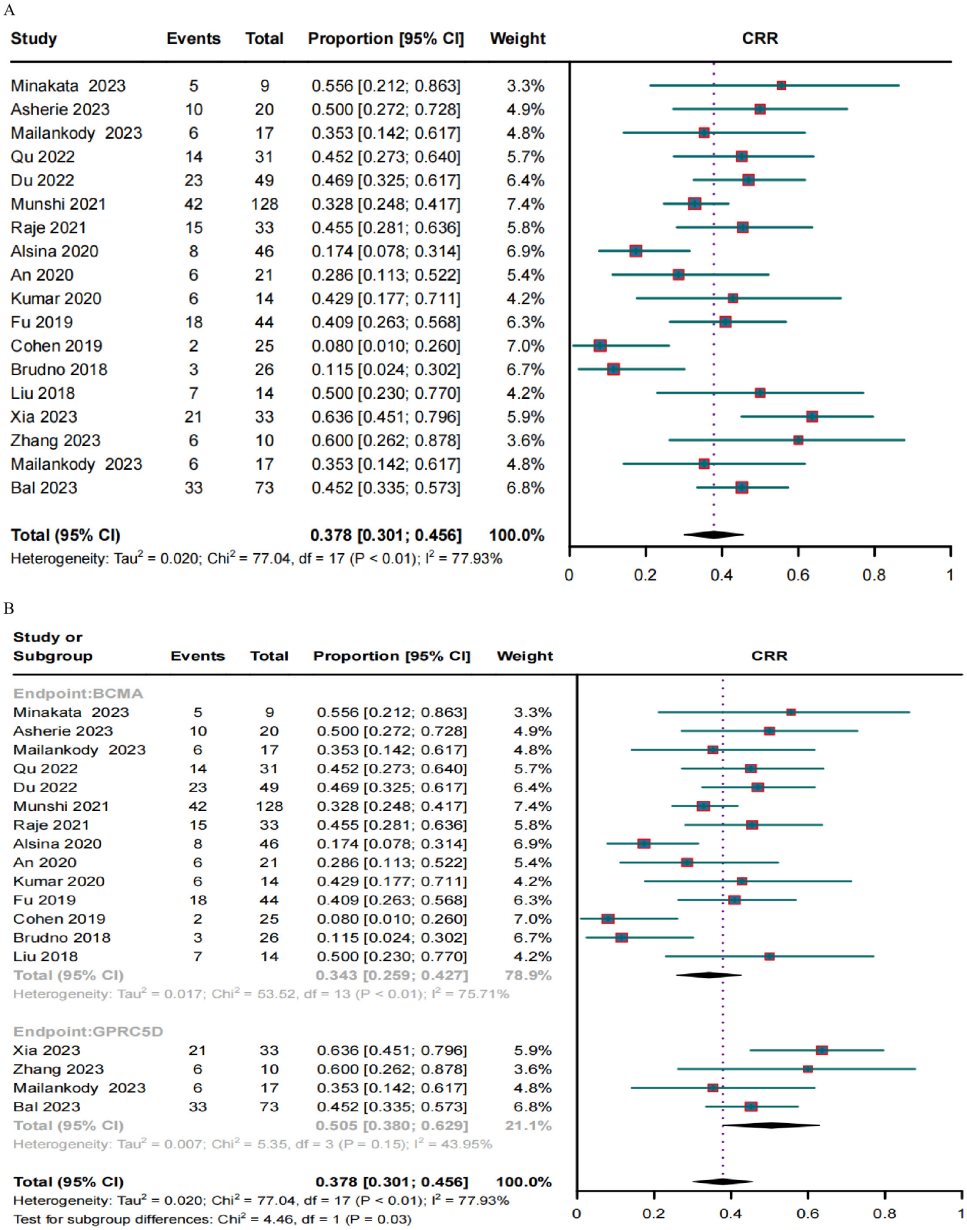
Forest plots of **(A)** pooled and **(B)** subgroup CRR data for BCMA and GPRC5D CAR-T.

A clinical response was evaluated in 636 patients. The pooled ORR and CRR were 79.1% (95% CI, 72.0%–86.3%; *I2* = 80.9%; *p* < 0.01; **Figure 2A**) and 37.8% (95% CI, 30.1%–45.6%; *I2* = 77.93%; *p* < 0.01; **Figure 3A**), respectively. In the subgroup analysis, BCMA and GPRC5D CAR-T demonstrated ORRs of 76.3% (95% CI, 67.9%–84.7%; **Figure 2B**) and 89.8% (95% CI, 82.8%–96.9%; **Figure 2B**), and CRRs of 34.3% (95% CI, 25.9%–42.7%; **Figure 3B**) and 50.5% (95% CI, 38.0%–62.9%; **Figure 3B**), respectively. Both the ORR and CRR were significantly higher for GPRC5D CAR-T than for BCMA CAR-T [*p* = 0.02 (**Figure 2B**) and 0.03 (**Figure 3B**), respectively].

### MRD negativity in RRMM patients treated with BCMA and GPRC5D CAR-T

**Figure 4:**
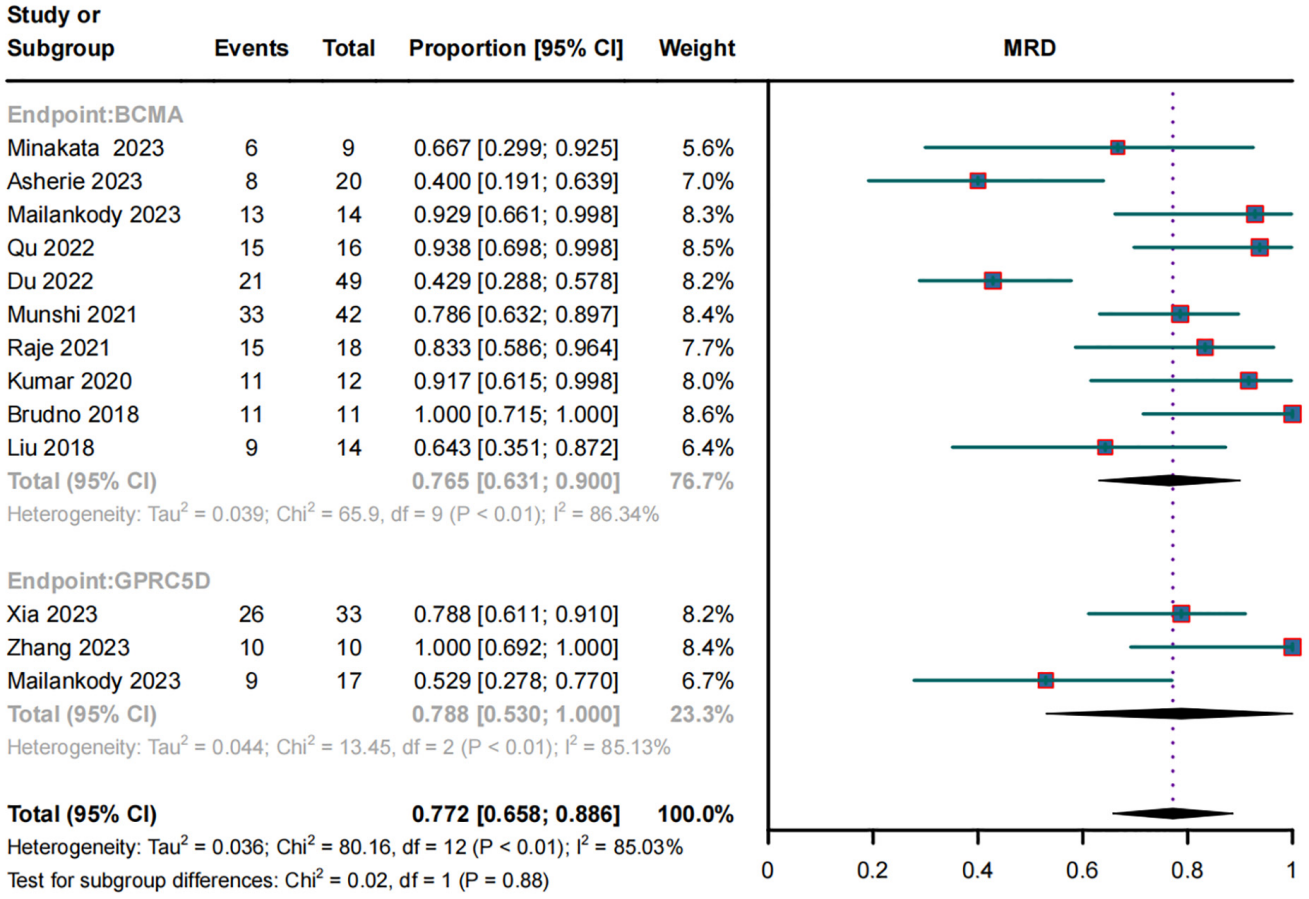
Forest plot of subgroup MRD negativity data for BCMA and GPRC5D CAR-T.

In the subgroup analysis of 10 BCMA CAR-T trials, 187 of 265 patients became MRD negative; their combined MRD-negativity rate was 77.2% (95% CI, 65.8%–88.6%; *I2* = 85.03%; *p* < 0.01; **Figure 4**). In the subgroup analysis of three GPRC5D CAR-T trials, 45 of 60 patients became MRD negative; their combined MRD negativity rate was 78.8% (95% CI, 53.0%–100%; **Figure 4**), which did not differ significantly from that for BCMA CAR-T.

### Relapse rates in RRMM patients treated with BCMA and GPRC5D CAR-T

**Figure 5:**
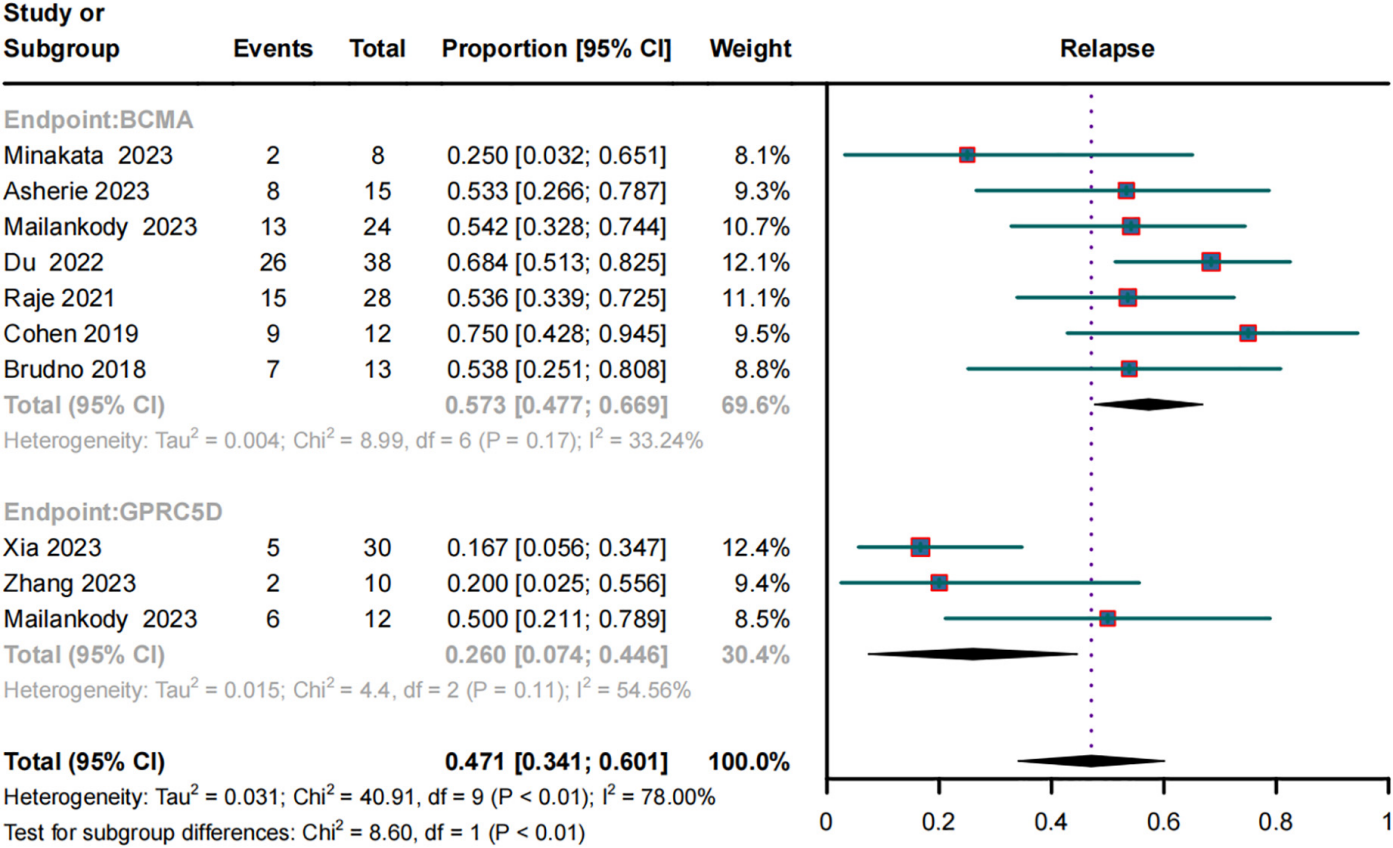
Forest plot of subgroup relapse rate data for BCMA and GPRC5D CAR-T.

Relapse rates were evaluated in seven BCMA CAR-T and three GPRC5D CAR-T trials. Their combined relapse rates were 57.3% (95% CI, 47.7%–66.9%) and 26.0% (95% CI, 7.4%–44.6%), respectively (**Figure 5**); the between-group difference in the rates was significantly different (*p* < 0.01).

### ORRs in RRMM patients with EMD or high-risk cytogenetic characteristics

**Figure 6:**
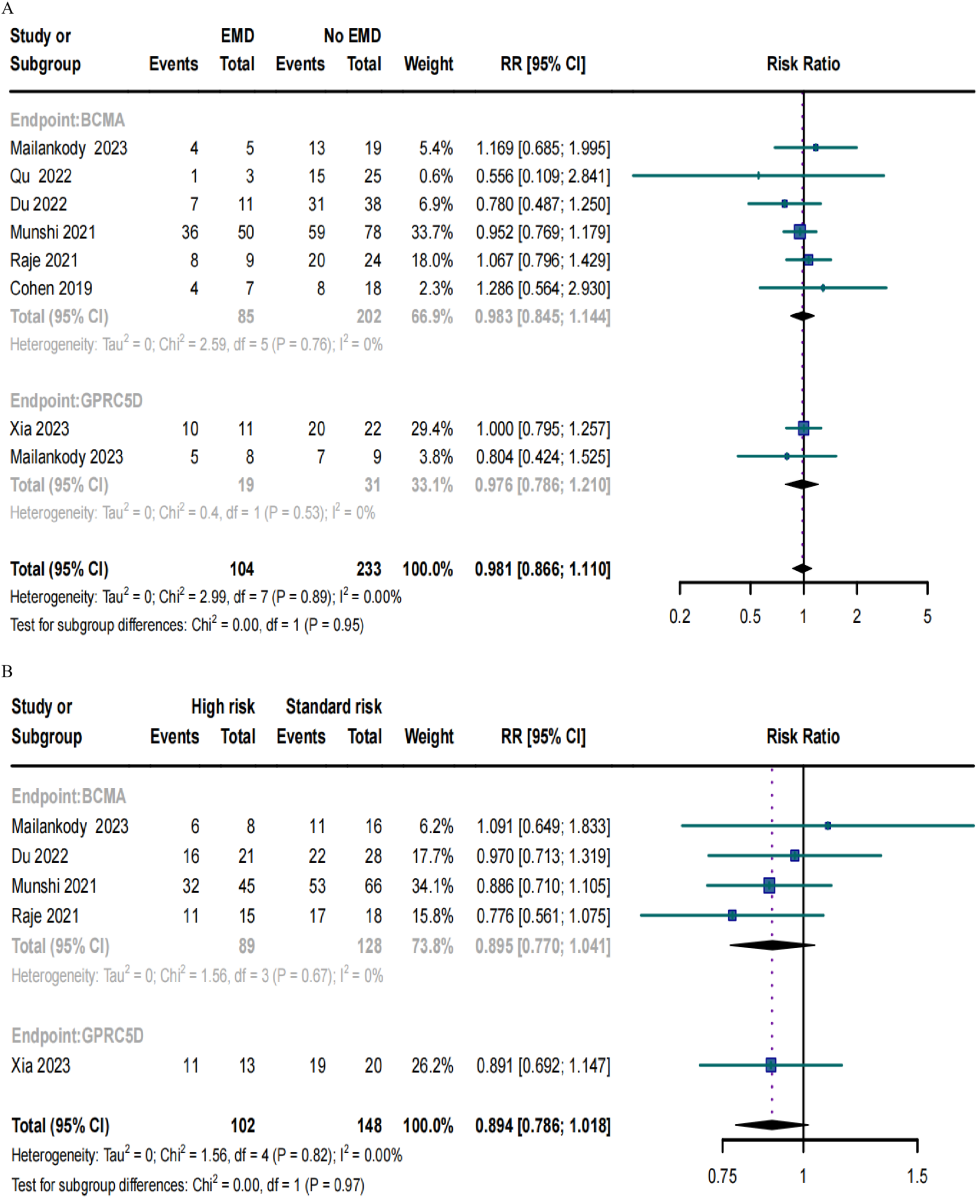
Forest plots of subgroup ORR data in **(A)** patients with or without EMD and **(B)** patients with or without high-risk cytogenetic characteristics.

Only eight trials included patients with EMD and demonstrated no significant differences in the ORRs for BCMA and GPRC5D CAR-T (*p* = 0.95; **Figure 6A**). Similarly, only five studies included patients with high cytogenetic characteristics, and their ORRs for BCMA and GPRC5D CAR-T did not demonstrate significant differences (*p* = 0.97; **Figure 6B**).

### Safety in RRMM patients treated with BCMA and GPRC5D CAR-T

**Figure 7:**
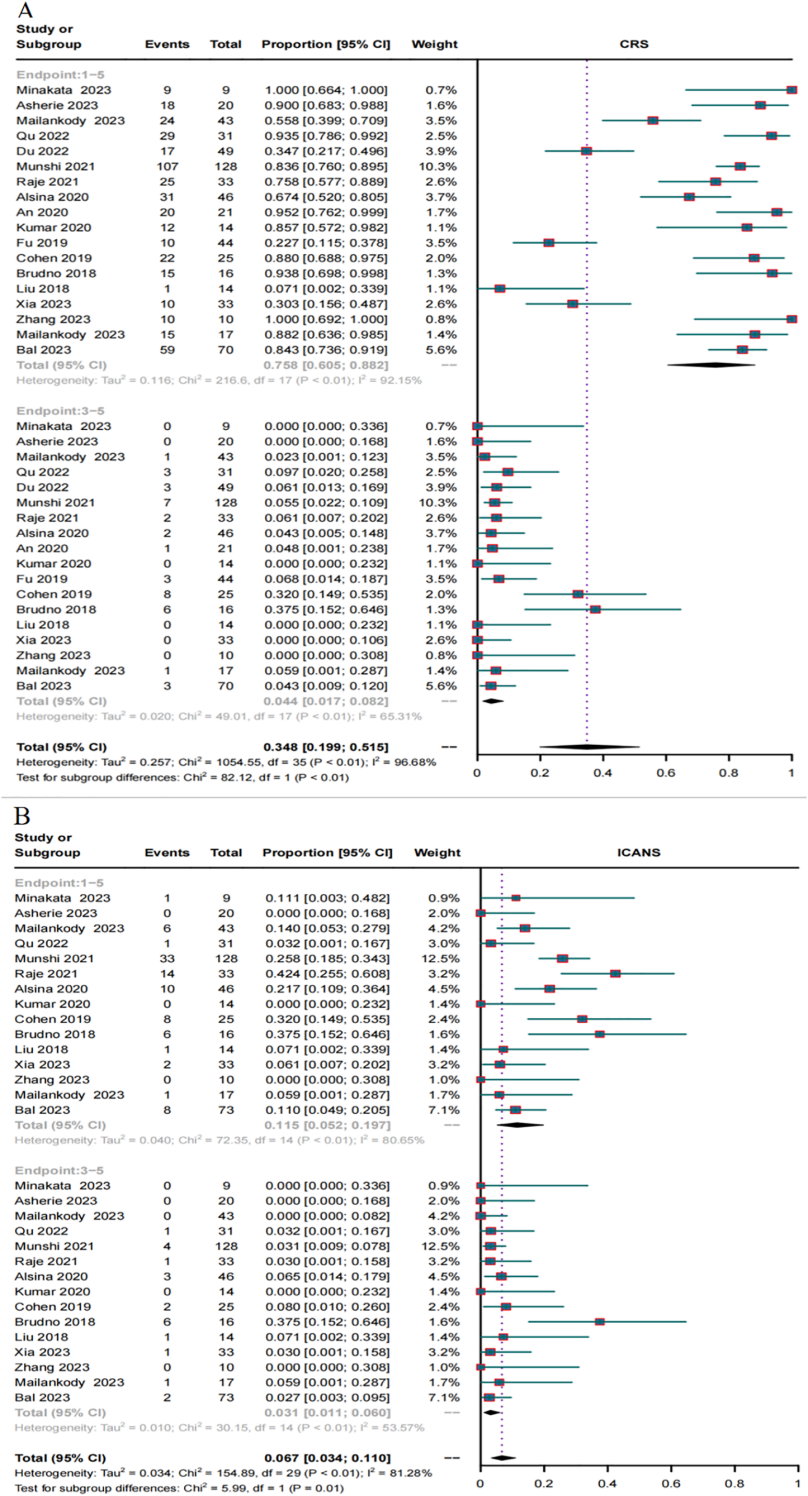
Forest plots of CRS and ICANS incidence stratified by severity. **(A)** Combined estimates for all CRS (1–5) and severity (3–5) grades. **(B)** Combined estimates for all ICANS (1–5) and severity (3–5) grades.

In total, 18 trials reported the overall CRS rate and 15 trials reported the overall ICANS rate. Additionally, 18 trials provided the severe CRS rate, and 15 trials provided the severe ICANS rate. The total CRS rate for BCMA and GPRC5D CAR-T was 75.8% (95% CI, 60.5%-88.2%), and the rate of severe (grade ≥3) CRS was 4.4% (95% CI, 1.7%–8.2%, **Figure 7A**). In turn, the total ICANS rate for both BCMA and GPRC5D CAR-T was 11.5% (95% CI, 5.2%–19.7%), and the rate of severe (grade ≥3) ICANS was 3.1% (95% CI, 1.1%–6.0%, **Figure 7B**).

**Figure 8:**
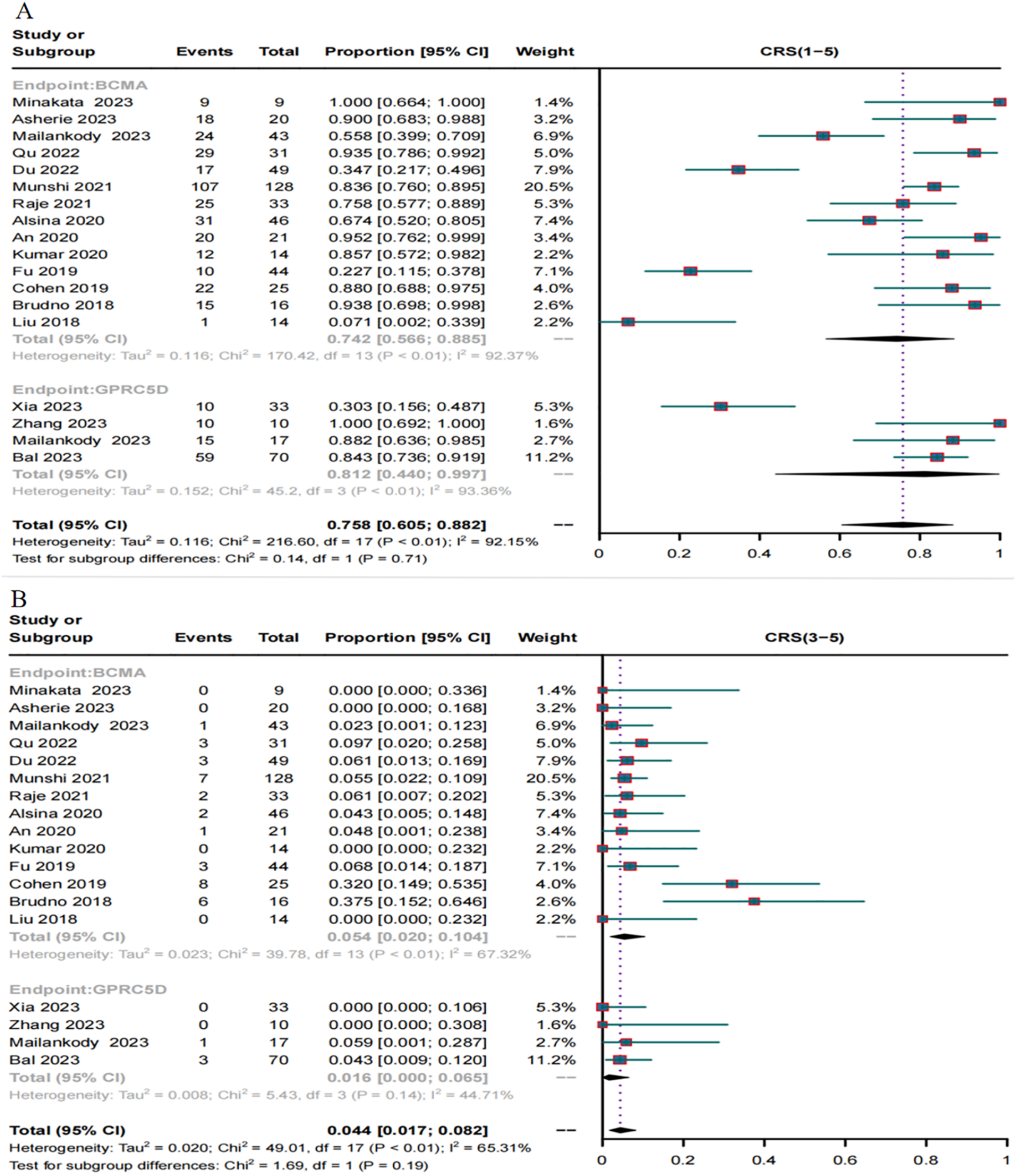
Forest map of the incidence of CRS. **(A)** Combined estimates for CRS grades 1-5. **(B)** Combined estimates for CRS severity (3–5) grades.

A total of 18 trials reported the incidence of CRS caused by BCMA CAR-T and GPRC5D CAR-T. The incidence of CRS grades 1-5 caused by BCMA CAR-T was 74.2% (95% CI, 56.6%–88.5%, **Figure 8A**), and the incidence of severe (≥ grade 3) CRS was 5.4% (95% CI, 2.0%–10.4%, **Figure 8B**). For GPRC5D CAR-T, the incidence of CRS grades 1–5 caused by GPRC5D CAR-T was 81.2% (95% CI, 44%–99.7%, **Figure 8A**), and the incidence of severe (≥ grade 3) CRS was 1.6% (95% CI, 0.0%–6.5%, **Figure 8B**).

**Figure 9:**
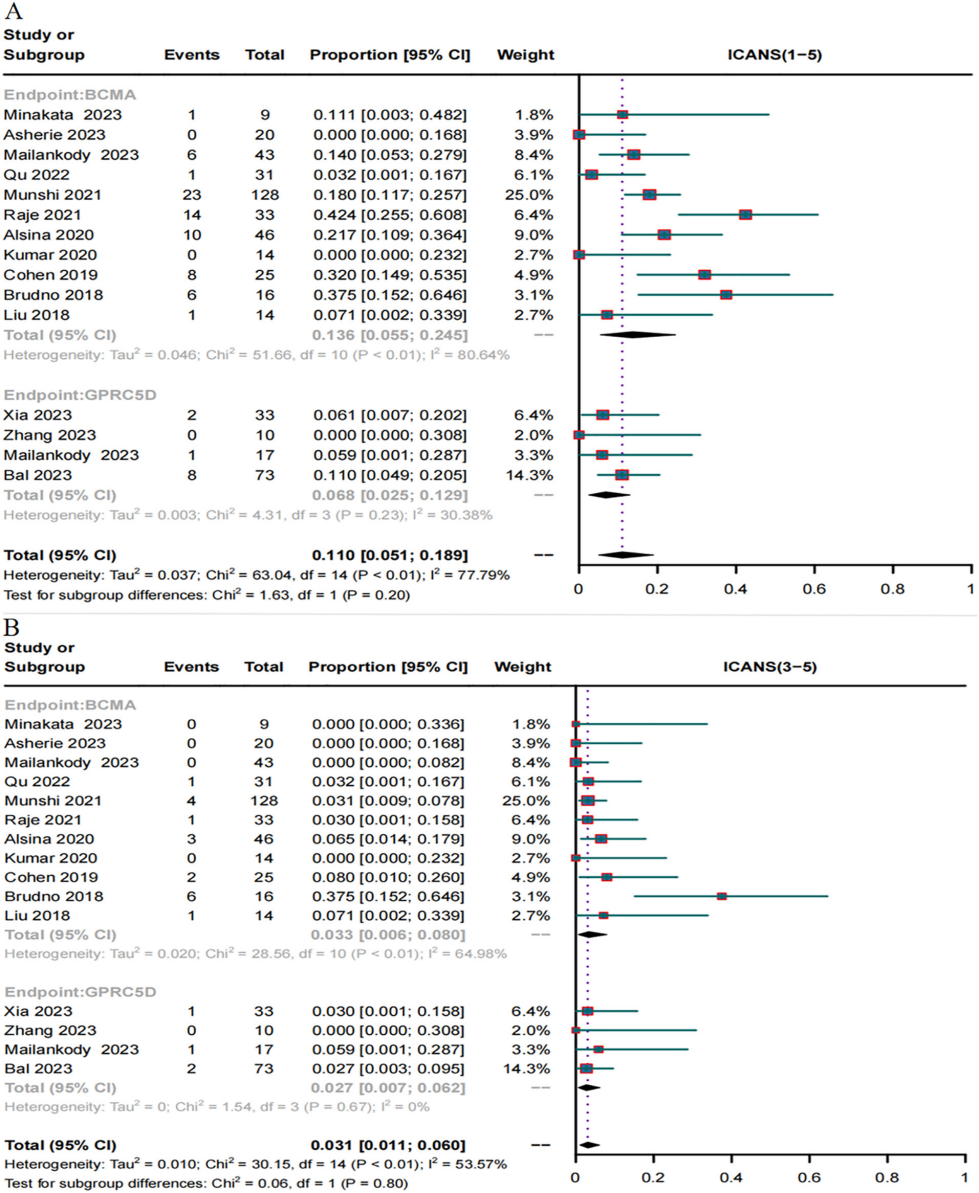
Forest map of incidence of ICANS. **(A)** Combined estimates for ICANS grades 1-5. **(B)** Combined estimates for ICANS severity (3–5) grades.

A total of 15 trials reported the incidence of ICANS caused by BCMA and GPRC5D CAR-T. The incidence of ICANS grades 1–5 caused by BCMA CAR-T was 13.6% (95% CI, 5.5%–24.5%, **Figure 9A**), while that of severe (≥ grade 3) ICANS was 3.3% (95% CI, 0.6%–8.0%, **Figure 9B**). In turn, for GPRC5D CAR-T, the incidence of ICANS grades 1-5 caused by GPRC5D CAR-T cell therapy was 6.8% (95% CI, 2.5%–12.9%, **Figure 9A**), and that of severe (≥ grade 3) ICANS was 2.7% (95% CI, 0.7%–6.2%, **Figure 9B**). The differences in the rates of adverse events (total or severe) for BCMA and GPRC5D CAR-T were insignificant (**Table 2**).

**Table 2:** Meta-regression CRS and ICANS.

Model	Predictor Variable	Estimate	SE	Z-Value	p-Value
All CRS (1-5 class)	Intercept CAR Target BCMA (vs. GPRC5D)	1.0886 0.1592	0.0784 0.1628	13.8799 0.9776	<0.0001 0.328
Serious CRS (3-5 class)	Intercept CAR Target BCMA (vs. GPRC5D)	0.2354 -0.1138	0.0452 0.0972	5.2104 -1.1706	<0.0001 0.2418
All ICANS (1-5 class)	Intercept CAR Target BCMA (vs. GPRC5D)	0.1519 -0.0921	0.0416 0.0730	3.6485 -1.2625	0.0003 0.2068
Serious ICANS(3-5 class)	Intercept CAR Target BCMA (vs. GPRC5D)	0.1827 -0.0228	0.0455 0.0884	4.0165 -0.2581	<0.0001 0.7963

SE, standard error.

### Risk of bias

We assessed the quality of evidence for the included studies using the GRADE system. The results thus obtained using the evidence from the GRADE system may be associated with some bias because all the included trials used a single-arm intervention design and demonstrated differences in follow-up duration (**Table 3**). Nevertheless, our estimated results were consistent, suggesting that they may be crucial for guiding clinical decisions and treatment.

**Table 3:** Grade recommendations.

Outcome	Group	No. Studies	No. Patients	Domains					Estimate [95% CI]	Quality of Evidence
				ROB	Imprecision	Inconsistency	Indirectness	Publication Bias		
ORR	BCMA GPRC5D	14 4	503 133	Serious	Moderate	Non-serious	Non-serious	Non-serious	0.763[0. 678-0.847] 0.898[0.828-0.969]	Low
CRR	BCMA GPRC5D	14 4	503 133	Serious	Moderate	Non-serious	Non-serious	Non-Serious	0.343[0.259-0.427] 0.505[0.380-0.629]	Low
MRD	BCMA GPR5D	10 3	205 60	Serious	Moderate	Non-serious	Non-serious	Non-serious	0.765[0.631-0.900] 0.788[0.530-1.000]	Low
Relapse	BCMA GPR5D	7 3	138 52	Serious	Moderate	Non-serious	Non-serious	Non-serious	0.573[0.477-0.669] 0.260[0.074-0.446]	Low
EMD	BCMA GPRC5D	6 2	287 50	Serious	Moderate	Non-serious	Non-serious	Non-serious	0.983[0.845-1.144] 0.976 [0.786-1.210]	Low
High-risk	BCMA GPRC5D	4 1	217 33	Serious	Moderate	Non-serious	Non-serious	Non-serious	0.814 [0.700-0.906] 0.044[0.017-0.082]	Low
CRS	Total Servre	17 18	622 636	Serious	Moderate	Non-serious	Non-serious	Non-Serious	0.054[0.020-0.104] 0.016[0.000-0.064]	Low
ICANS	Total Servre	13 15	522 552	Serious	Moderate	Non-serious	Non-serious	Non-serious	0.104[0.042-0.190] 0.031[0.011-0.060]	Low

BCMA, B cell membrane antigen; GPRC5D, G protein-coupled receptor, class C group 5 member D; ORR, overall response rate; CRR, complete response rate; MRD, minimal residual lesion negative; EMD, extramedullary disease; CRS, cytokine release syndrome; ICANS, immune effector cell-associated neurotoxicity syndrome; ROB, risk of bias.

## Discussion

Clinically, the treatment of RRMM remains difficult and warrants further development (42). Cellular immunotherapy may lead to effective outcomes in patients with RRMM (43). Several recent studies have assessed the efficacy and safety of BCMA CAR-T in RRMM (44, 45). BCMA is considered critical for the survival of bone marrow plasma cells (46). However, MM patients with negative or low BCMA expression will still relapse after receiving BCMA-targeted CAR T-cell therapy, with problems arising from immune escape (11, 31, 36). Moreover, although considered a rare event, BCMA antigen loss may occur after anti-BCMA treatment due to biallelic deletion of the BCMA locus on chromosome 16 or reversible downregulation of BCMA expression, which prevents subsequent response to BCMA-targeted therapy (47–50). Therefore, identifying more specific or consistent MM targets may help mitigate BCMA escape-mediated recurrence. GPRC5D is a target validated for rationally designed immunotherapeutic strategies because it is preferentially expressed on plasma cells; a preclinical study demonstrated its efficacy in a BCMA escape model (19). Compared with BCMA, GPRC5D has better specificity, and its expression does not decrease with time. Furthermore, they are independently expressed and they can be single- or double-targeted to develop therapeutic drugs (51). However, the efficacy and safety of GPRC5D CAR-T in patients with RRMM warrants evaluation.

To our knowledge, this is the first systematic review and meta-analysis comparing BCMA and GPRC5D CAR-T outcomes in patients with RRMM. BCMA-targeted CAR-T cell therapy has shown effectiveness in RRMM patients, but there are problems of relapse and antigen escape in patients who are BCMA-negative or have a low expression (52). As a target of immunotherapy, GPRC5D has shown potential efficacy in BCMA escape models, and its expression has better specificity and persistence (19, 53). Our subgroup analysis also showed that GPRC5D CAR-T had a higher MRD negative rate (78.8%) than BCMA CAR-T (76.5%). As mentioned above, GPRC5D has BCMA-independent expression on plasma cells, ensuring continued expression even after BCMA relapse. Studies have shown that the largest population of CD138^+^ cells express both BCMA and GPRC5D. However, GPRC5D expression is dominant and an independent expression pattern targeting a second antigen (GPRC5D) may increase the frequency, depth, and/or duration of response in patients with BCMA-low or -negative MM plasma cells (13, 19). Our study showed in turn a significantly higher CRR for GPRC5D CAR-T (50.5% vs. 34.3%) than BCMA CAR-T when treating RRMM. GPRC5D is a c7-transmembrane receptor protein that is, unlike BCMA, not easily shed in serum. Thus, targeting GPRC5D is less likely to cause an “antigen-sinking” effect that would reduce subsequent CAR-T efficacy (54, 55). Similarly, in our subgroup analysis, GPRC5D CAR-T achieved a significantly higher ORR (89.8%) than BCMA CAR-T (76.3%).

Studies have reported that BCMA CAR-T does not yield stable survival in patients with MM, with most patients eventually demonstrating relapse (8–11). In this study, the combined relapse rate of BCMA CAR-T was high (57.3%), whereas that of GPRC5D CAR-T was much lower (26.0%)—suggesting that GPRC5D CAR-T is associated with lower rates of relapse in patients with RRMM. High-risk cytogenetic characteristics and EMD are risk factors for poor MM prognosis (4, 56). When comparing the efficacy of BCMA and GPRC5D CAR-T in patients with and without these adverse prognostic factors, no differences in ORR were noted between patients for either of the modalities. This suggests that both GPRC5D CAR-T and BCMA CAR-T can alleviate the poor outcomes associated with high-risk karyotypes in MM patients.

BCMA is highly expressed in myeloma cells but shows limited expression in normal tissues and B cells. GPRC5D is highly expressed on the surface of myeloma cells, while its expression in normal tissues is limited to the hair follicle region. It has been reported that GPRC5D-targeted CAR-T cell therapy can cause a low degree of skin and nail toxicity and oral adverse events, while cerebellar toxicity has been reported in only two MM cases (19, 20). The adverse reactions caused by CAR-T cell therapy are mainly CRS and ICANS, and there is little literature data on the incidence of other adverse reactions caused by GPRC5D CAR-T. Therefore, CRS and ICANS were analyzed in this study to evaluate the safety of the two cell therapies. Studies have shown that due to the short extracellular domain, epitopes exposed by GPRC5D for T cell redirection agents may be closer to the plasma membrane. This in turn promotes tighter immune synapses between T cells and target cells, which may confer more significant cytotoxicity (20, 57). This study also found that GPRC5D CAR-T caused a higher incidence of CRS grades 1-5 than BCMA CAR-T (81.2% vs. 74.2%), although in the subgroup analysis, this difference was not statistically significant. In contrast, a significantly lower incidence of ICANS grades 1-5 (6.8% vs. 13.6%), ICANS grades 3-5 (2.7% vs. 3.3%), and CRS grades 3-5 (1.6% vs. 5.4%) was observed for GPRC5D CAR-T compared to BCMA CAR-T. Therefore, the incidence of CRS and ICANS grades 3-5 in both cell therapies is acceptable.

In summary, our results indicated that GPRC5D CAR-T can induce a substantial response in patients with RRMM. However, research on GPRC5D CAR-T for RRMM is in its early stages, hence further studies elucidating its mechanisms are warranted. In particular, phase 2 and 3 clinical trials are required to focus on the efficacy and safety of GPRC5D CAR-T in specific subgroups to guide its applicability for individualized treatments. In the current study, we minimized the effects of heterogeneity by using a random-effects model and evaluated the quality of evidence using the GRADE system. Because most of the included trials on GPRC5D CAR-T used a short follow-up duration and because many clinical trials on GPRC5D CAR-T are ongoing, we did not analyze overall and progression-free survival.

## Conclusions

In patients with RRMM, GPRC5D-targeted CAR-T cell therapy may demonstrate superior efficacy compared to BCMA-targeted CAR-T cell therapy. Consequently, GPRC5D could represent a more promising alternative therapeutic target for RRMM patients, particularly those who have experienced relapse following BCMA CAR-T treatment. Beyond offering new avenues for future research, our findings may assist healthcare professionals in making evidence-based clinical decisions and providing optimal treatment options for individuals with RRMM.

## Data Availability

The original contributions presented in the study are included in the article/supplementary material. Further inquiries can be directed to the corresponding authors.
